# Abnormal Fetal/Neonatal Lung Development Manifested as Some Functional Heart Abnormalities During the Third Trimester of Fetal Life

**DOI:** 10.3390/biomedicines13102324

**Published:** 2025-09-23

**Authors:** Julia Murlewska, Oskar Sylwestrzak, Maciej Słodki, Iwona Strzelecka, Łukasz Sokołowski, Monika Wójtowicz-Marzec, Iwona Maroszyńska, Ewa Cichos, Hanna Romanowicz, Anita J. Moon-Grady, Maria Respondek-Liberska

**Affiliations:** 1 Department of Prenatal Cardiology, Polish Mother’s Memorial Hospital Research Institute, 93-338 Lodz, Poland; maciejslodki@op.pl (M.S.); i.j.strzelecka@gmail.com (I.S.); l.sokolowski90@gmail.com (Ł.S.); maria.respondek-liberska@uni.lodz.pl (M.R.-L.); 2 Department of Obstetrics and Gynecology, Polish Mother’s Memorial Hospital Research Institute, 93-338 Lodz, Poland; 3 Medicine Faculty, Mazovian Academy in Plock, 09-402 Plock, Poland; 4 Department of Diagnoses and Prevention of Fetal Malformations, Medical University of Lodz, 90-419 Lodz, Poland; 5 Chair and Department of Paediatric Nursing, Faculty of Health Sciences, Medical University of Lublin, 20-059 Lublin, Poland; monikawmarzec@gmail.com; 6 Department of Neonatal Intensive Care and Congenital Malformations of Newborns and Infants, Polish Mother’s Memorial Hospital Research Institute, 93-338 Lodz, Poland; iwona.maroszynska@iczmp.edu.pl; 7 Department of Clinical Pathomorphology, Polish Mother’s Memorial Hospital Research Institute, 93-338 Lodz, Poland; cichosewa@iczmp.edu.pl (E.C.); hanna-romanowicz@wp.pl (H.R.); 8 Division of Pediatric Cardiology, Benioff Children’s Hospital, University of California, San Francisco, CA 94158, USA; anita.moongrady@ucsf.edu; 9 Fetal Treatment Center, Benioff Children’s Hospital, University of California, San Francisco, CA 94158, USA

**Keywords:** pulmonary hypertension, fetal echocardiography, coarctation of the aorta, congenital heart disease, prenatal diagnosis, neonatal outcomes

## Abstract

**Background**: Pulmonary hypertension (PH) in newborns is a rare but serious condition and potentially life-threatening disorder, often initially confused with congenital heart disease due to overlapping echocardiographic findings in the late third trimester. Evidence on prenatal predictors of postnatal PH is limited. We aimed to describe detailed third-trimester echocardiographic findings associated with postnatal PH in infants with prenatally suspected CoA based on a retrospective case series. **Methods**: We reviewed 18 years of fetal echocardiography (2004–2022) in a tertiary maternal–fetal–neonatal center. We identified fetuses with suspected coarctation of the aorta (CoA) in late gestation who were delivered at term (≥37 weeks) and had prolonged neonatal hospitalization (>10 days) without cardiac surgery or catheterization. Z-scores for cardiac dimensions were calculated. All examinations were performed by experienced fetal cardiologists. Postnatal evaluations confirmed PH based on echocardiographic and clinical findings. **Results**: Among 19,836 fetuses examined, 138 were prenatally suspected of CoA. In 70 cases, this diagnosis was not confirmed postnatally (false positives). Of these, eight infants (0.04% of the total cohort) developed postnatal PH. Postnatally, all eight neonates required intensive care. Prenatal features included ventricular/atrial disproportion (7/8), cardiomegaly (8/8), main pulmonary artery dilatation (10.2 ± 2.2 mm; Z-score +2.7 ± 1.3), tricuspid regurgitation (8/8), pulmonary regurgitation (4/8), and interventricular septal hypertrophy (>4.5 mm in 5/8). Postnatal evaluations confirmed PH based on echocardiographic criteria (elevated right ventricular pressure, septal flattening/bowing, right ventricular dilation or dysfunction, and abnormal shunt direction) combined with clinical compromise. All infants received prostaglandin E1 (PGE1) initially; none required extracorporeal membrane oxygenation-ECMO. Three died, while five survived with medical management (oxygen, inhaled nitric oxide, sildenafil). **Conclusions**: Specific functional abnormalities on late third-trimester echocardiography may indicate impaired pulmonary vascular adaptation and predict postnatal PH, particularly in cases initially suspected of CoA. Recognition and awareness of these findings can guide delivery planning, neonatal surveillance, and timely intervention. Prospective multicenter studies are needed to validate these associations and refine prenatal screening protocols.

## 1. Introduction

Pulmonary hypertension (PH) of the newborn, more specifically persistent pulmonary hypertension of the newborn (PPHN), remains one of the most challenging disorders in neonatal medicine. Characterized by failure of the normal circulatory transition after birth, PH is marked by sustained elevation of pulmonary vascular resistance (PVR), right-to-left shunting through the ductus arteriosus (DA) or foramen ovale (FO), and resulting systemic hypoxemia. Despite advances in neonatal intensive care, including the use of mechanical ventilation, inhaled nitric oxide (iNO), and extracorporeal membrane oxygenation (ECMO), the condition is associated with significant morbidity and mortality [1].

### 1.1. Historical Background

The concept of persistent fetal circulation was first described by William Harvey in 1628, who noted the unique fetal cardiovascular pathways that allow blood to bypass the lungs in utero. In the 1960s, the clinical recognition of PPHN became clearer with the identification of sustained right-to-left shunting in neonates suffering from respiratory distress syndrome and meconium aspiration. Management strategies have evolved over time: hyperventilation and alkalinization were common in the 1980s, later supplemented by pharmacologic vasodilators such as tolazoline, and more recently by iNO and sildenafil. Nevertheless, mortality rates for severe PH remain between 10 and 20%, with survivors at risk of long-term complications, including chronic lung disease and neurodevelopmental impairment [1].

### 1.2. Pathophysiology of Neonatal Pulmonary Hypertension

In normal physiology, the fetal pulmonary circulation is characterized by high resistance and low blood flow, with oxygenated blood shunted from the placenta to the systemic circulation via the ductus venosus, foramen ovale, and ductus arteriosus. At birth, the first breaths trigger a rapid decrease in PVR, allowing pulmonary blood flow to rise and facilitating the transition to postnatal circulation. In PH, this drop fails to occur or is reversed due to structural vascular remodeling, parenchymal lung disease, meconium aspiration, or abnormal pulmonary vascular tone. The persistent elevation of pulmonary artery pressure (PAP) imposes strain on the right ventricle (RV), leading to hypertrophy, tricuspid regurgitation, and potentially heart failure. Emerging data suggest that some functional cardiac changes detectable in late gestation may represent early markers of impaired pulmonary vascular development, even when no structural congenital heart disease is present. These include RV and right atrial dilation, increased MPA diameter, abnormal shunting, and septal bowing [2,3,4,5,6,7,8,9,10,11,12,13,14,15,16,17,18,19,20,21,22,23,24,25,26,27,28,29,30,31].

### 1.3. Epidemiology and Clinical Impact

The incidence of PH is estimated at 1.9 per 1000 live births, though rates vary geographically and are influenced by perinatal care practices [9,18]. Risk factors include maternal diabetes, pre-eclampsia, obesity, smoking, and antenatal drug exposure (e.g., selective serotonin reuptake inhibitors, non-steroidal anti-inflammatory drugs) [8,9,14,19]. Fetal conditions such as congenital diaphragmatic hernia, meconium aspiration syndrome, meconium peritonitis, restrictive foramen ovale, and certain cardiac malformations have also been implicated [2,4,6,22].

Despite this knowledge, the prenatal detection of PH remains uncommon. Most diagnoses are established postnatally, typically when infants present with hypoxemia that is disproportionate to lung disease severity. This diagnostic delay can lead to suboptimal management, delayed initiation of iNO, and a higher risk of morbidity [2,3,4,5,6,7,8,9,10,11,12,13,14,15,16,17,18,19,20,21,22,23,24,25,26,27,28,29,30,31].

### 1.4. Rationale for Study

Although fetal echocardiography is widely used for diagnosing congenital heart defects, its role in predicting functional cardiopulmonary disorders such as PH has been less thoroughly studied. Our tertiary center, with 18 years of fetal echocardiographic data, offered a unique opportunity to retrospectively identify cases where prenatal suspicion of CoA in the third trimester ultimately corresponded to postnatal PH rather than structural aortic obstruction. By characterizing prenatal echocardiographic features and linking them to neonatal outcomes, we aimed to provide additional evidence to guide obstetricians, neonatologists, and pediatric cardiologists in early detection and management of this rare but serious condition.

## 2. Materials and Methods

### 2.1. Study Design and Setting

This study was conducted as a retrospective observational analysis at the Department of Prenatal Cardiology, Polish Mother’s Memorial Hospital Research Institute in Łódź, Poland, a tertiary referral center serving a large regional population. The study period extended from January 2004 to December 2022. Over this time, the center performed 19,836 fetal echocardiographic examinations, creating a comprehensive database of prenatal cardiovascular assessments and linked neonatal outcomes.

All patients consented to the use of their data for scientific analysis. Because this study focused on the interpretation of previously collected data, no additional approval from the local ethics committee was required.

### 2.2. Inclusion and Exclusion Criteria

We included fetuses who met the following criteria:

- Underwent detailed third-trimester echocardiography (≥37 weeks gestation);
- Had a prenatal suspicion of coarctation of the aorta (CoA);
- Required neonatal hospitalization exceeding 10 days, without undergoing cardiac surgery or catheterization in the neonatal period.

Exclusion criteria were as follows:

- Lack of complete prenatal or postnatal records;
- Cases with confirmed structural congenital heart disease including true CoA;
- Cases with severe extracardiac anomalies leading to neonatal death unrelated to PH.

### 2.3. Echocardiographic Protocol

Fetal echocardiography was performed using high-resolution ultrasound systems (various generations of GE Voluson and Philips platforms across the study period). Standardized protocols were followed, including acquisition of the following:

- Four-chamber view (for ventricular and atrial dimensions, heart-to-chest ratio).
- Outflow tract views (right and left ventricular outflow tracts).
- Three-vessel and trachea (3VT) view (for comparison of aortic and pulmonary artery diameters).
- Doppler assessments of tricuspid and pulmonary regurgitation, ductus arteriosus flow velocity, and pulmonary artery acceleration time (PAT).

Cardiac dimensions were indexed to gestational age using the Schneider Z-score method [32]. Cardiomegaly was defined as a heart area-to-chest area (HA/CA) ratio > 0.36. Interventricular septal hypertrophy was defined as IVS thickness > 4.5 mm in the third trimester. Disproportion was defined qualitatively by an enlarged right atrium or right ventricle relative to the left, and by an increased MPA/Ao ratio (>1.3 considered abnormal) (Table 1).

### 2.4. Data Collection and Analysis

Postnatal data were retrieved from hospital records, (Table 2), including the following information:

- Gestational age at delivery and birth weight;
- Neonatal echocardiography findings right ventricular systolic pressure-RVSP, pulmonary artery pressure-PAP, septal position, shunt direction, valve regurgitation);
- Respiratory support requirements (oxygen therapy, continuous positive airway pressure-CPAP, mechanical ventilation, iNO);
- Other medical therapies (e.g., sildenafil, milrinone);
- Duration of neonatal hospitalization;
- Survival and postmortem findings when applicable.

Given the small number of PH cases, statistical analysis was primarily descriptive. Continuous variables are reported as the mean ± standard deviation (SD). Categorical variables are presented as frequencies and percentages. Statistical analyses were performed using Statistica 13.1 (StatSoft, Tulsa, OK, USA) and Microsoft Excel 365.

## 3. Results

### 3.1. Cohort Description

**Figure 1:**
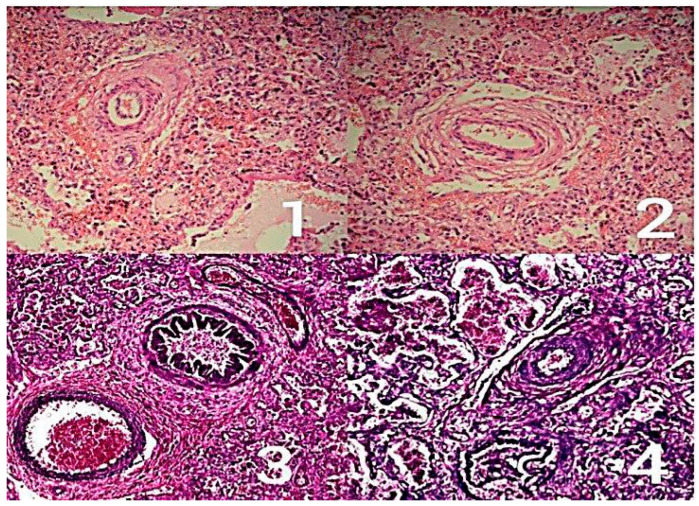
Pulmonary hypertension-courtesy of Pathomorphology Department Polish Mother Memorial Hospital Research Institute, Lodz, Poland. 1—pulmonary hypertension, H + E magnification 180×. Concentric thickening of the intrapulmonary artery wall. 2—pulmonary hypertension, H + E magnification 180×. Concentric thickening of the walls of the intrapulmonary artery and vein. 3—pulmonary hypertension, incl. elastic staining with magnification 180×. Concentric thickening of the walls of the intrapulmonary artery and vein with splitting of elastic fibers. 4—pulmonary hypertension, elastic staining for the presence of elastic fibers, with magnification 180×. Concentric thickening of the intra-pulmonary artery wall with a splice of elastic fibers.

From the total 19,836 fetuses evaluated over the study period, 138 (0.7%) were suspected prenatally of having CoA. Of these, 68 (49.3%) were confirmed postnatally (Figure 1 shows histopathologic findings of PH), while 70 (50.7%) represented false positive diagnoses. Within the false positive group, eight infants (11.4% of false positives; 0.04% of total cohort) were subsequently diagnosed with postnatal pulmonary hypertension (PH), accompanied by a complicated neonatal course. These eight cases formed the study group.

The mean gestational age (GA) at prenatal diagnosis was 33.4 ± 1.86 weeks, with values ranging from 31 to 37 weeks. The mean heart area-to-chest area (HA/CA) ratio was 0.43 ± 0.03, above the threshold for cardiomegaly in all eight cases.

### 3.2. Prenatal Echocardiographic Findings

The principal prenatal findings in the eight PH cases are summarized in Table 1. Key observations included the following:

- Ventricular disproportion: observed in seven out of eight cases, typically with right-sided dominance.
- Atrial disproportion: present in six out of eight cases, also favoring the right atrium.
- Three-vessel view (3VV) disproportion: evident in all eight out of eight cases, with a consistently enlarged pulmonary artery compared to the aorta.
- Main pulmonary artery (MPA) dilation: a mean diameter of 10.2 ± 2.2 mm, corresponding to a Z-score of +2.7 ± 1.3, indicating significant enlargement.
- Valve regurgitation: all fetuses showed tricuspid regurgitation with velocities ranging from 2.0 to 3.6 m/s; four out of eight had concomitant pulmonary regurgitation.
- Interventricular septal hypertrophy: identified in five out of eight cases, with IVS > 4.5 mm.
- Aortic isthmus narrowing: present in several fetuses, raising suspicion of CoA prenatally, although this was not confirmed postnatally.
- Ductus arteriosus Doppler: flows were generally patent, with mildly elevated velocities (mean 142 ± 14 cm/s) but without definitive restriction.

Taken together, the constellation of findings suggested right-sided pressure overload and abnormal pulmonary circulation already in utero.

### 3.3. Postnatal Outcomes

All eight neonates were delivered at a mean gestational age of 37 ± 1.1 weeks, with a mean birth weight 3195 ± 475 g. Despite the absence of structural heart disease, all presented with cardiopulmonary compromise shortly after birth.

- Day 1: All eight infants demonstrated echocardiographic signs suggestive of CoA (narrowed aortic isthmus, right-sided dilation, and abnormal DA flow). Each received prostaglandin E1 (0.01 μg/kg/min for 3–5 days) to maintain ductal patency. None developed severe hypoxemia requiring immediate ECMO.
- Subsequent course: Serial echocardiography revealed regression of tricuspid regurgitation, interventricular septal hypertrophy, and MPA dilation in survivors. Shunting across the FO and DA normalized as pulmonary pressures gradually decreased.
- Therapies: All infants required supplemental oxygen and CPAP; three received inhaled nitric oxide, and two additionally received sildenafil. The mean duration of hospitalization was 25 ± 11 days (range: 13–50 days).
- Outcomes: Three neonates died-one with confirmed trisomy 18, one with necrotizing enterocolitis (NEC), and one with severe cardiopulmonary compromise. Five infants survived, all discharged home after improvements in PH with medical management (Table 2).

### 3.4. Comorbidities

Notable comorbidities included the following:

- Trisomy 18 (Case 4): The only chromosomal anomaly in the cohort; this infant also had PH and did not survive.
- Bilateral superior vena cava (Case 8): Associated with prolonged hospital stay (50 days) and NEC.
- Meconium peritonitis and abdominal distension (Case 6): This infant died postnatally despite colostomy.
- Meconium aspiration syndrome (Case 5): Complicated the neonatal course but the infant survived.

These findings suggest that fetal cardiac abnormalities associated with suspected CoA may coincide with placental or neonatal comorbidities, amplifying the risk of PH.

## 4. Discussion

### 4.1. Interpretation of Findings

Our case series highlights that functional right-sided abnormalities detected on third-trimester fetal echocardiography, including cardiomegaly, right atrial and ventricular enlargement, MPA dilation with elevated Z-scores, tricuspid regurgitation, and interventricular septal hypertrophy, were recurrent features among fetuses who later developed postnatal PH despite not having true CoA. These findings suggest that increased pulmonary vascular resistance in utero may generate hemodynamic adaptations visible before birth, foreshadowing maladaptive postnatal transition.

Importantly, in all eight cases, initial postnatal echocardiography suggested CoA, leading to the initiation of PGE1 therapy. Only serial postnatal imaging clarified the absence of structural aortic obstruction, revealing that the underlying pathology was functional PH. This underscores the diagnostic overlap between late prenatal suspicion of CoA and postnatal PH, a distinction with major implications for clinical management.

### 4.2. Placental and Maternal Factors

Our analysis did not reveal maternal diabetes, pre-eclampsia, steroid use, or smoking in the eight affected pregnancies, though these are well-established contributors to neonatal PH [8,9,14,20]. Previous studies highlight maternal vascular underperfusion and placental pathologies (e.g., distal villous hypoplasia, fibrinoid necrosis) as significant contributors to impaired pulmonary vascular development [20]. Although placental histology was available in some of our cases, systematic assessment of maternal diet and environmental exposures was not performed, limiting our ability to evaluate these influences.

Nonetheless, the observed association between fetal right-sided cardiac strain and postnatal PH raises the possibility that subtle, undetected maternal or placental factors may have contributed. For instance, reduced placental efficiency could increase fetal pulmonary afterload, producing the observed MPA dilation and ventricular disproportion (Table 3).

### 4.3. Comparison with Previous Studies

There is sparse research on prenatal predictors of neonatal PH. Earlier reports, including those from our center, have described cases of PH in association with hypoplastic left heart syndrome, transposition of the great arteries, and congenital diaphragmatic hernia [21,22]. The study by van Nisselrooij et al. (2018) [24] focused on isolated ventricular disproportion without confirmed CoA and found a subset of neonates developing PH postnatally. Our findings are broadly consistent, but we add granularity by describing quantitative MPA Z-scores, septal hypertrophy, and valve regurgitation, which may refine risk stratification (Table 4).

Recent reports have also emphasized fetal pulmonary artery stiffness, altered pulmonary venous Doppler patterns [19], and maternal factors such as vitamin D deficiency [14] and SSRI exposure [8,9]. Taken together, the emerging evidence points to a multifactorial etiology where prenatal cardiovascular findings intersect with maternal and placental influences to shape neonatal outcomes (Table 3).

### 4.4. Clinical Implications

Early recognition of these prenatal signs [25,26,27,28,29,30,31] (Table 5) may prompt closer neonatal monitoring and timely initiation of therapies such as oxygen supplementation, inhaled nitric oxide, or sildenafil. Awareness of these features can reduce delays in PH diagnosis and treatment.

### 4.5. Limitations and Future Directions

This study is limited by its retrospective design and the very small number of PH cases. Only descriptive statistics were feasible, and confidence intervals could not be reliably estimated. Inter-observer variability was minimized by using experienced operators, but not formally quantified. Future multicenter prospective studies are needed to validate these findings and explore additional prenatal markers, including pulmonary venous Doppler assessment.

## 5. Conclusions

Third-trimester fetal echocardiography can reveal functional cardiac abnormalities that may predict postnatal PH, particularly in cases with false positive suspicion of CoA. Recognizing these features could support timely neonatal care and potentially improve outcomes.

## Figures and Tables

**Table 1:** Quality data of prenatal echocardiographic findings and hospital stay. GA—gestational age at prenatal diagnosis, HA/CA—heart area/chest area, MPA—main pulmonary artery, Ao—aorta, DA—ductus arteriosus, and SD—standard deviation.

	Case 1	Case 2	Case 3	Case 4	Case 5	Case 6	Case 7	Case 8	Mean	SD
GA [weeks] at prenatal ECHO	33	31	32	33	33	32.4	35	37.4	33.4	+/−1.86
HA/CA	0.48	0.46	0.4	0.46	0.45	0.4	0.4	0.4	0.43	+/−0.03
Heart width [mm]	38	49	42	42	42	42	41	37	41.6	+/−3.4
MPA [mm]	10	7	7.5	11.6	10	13	13	9.3	10.2	+/−2.2
MPA Z-scores	+3.47	+1.26	+1.49	+4.42	+3.47	+3.77	+3.19	−0.9	+2.7	+/−1.3
Ao [mm]	5	5	5.5	7.8	5	5	6	5.2	5.6	+/−0.9
Ao Z-score	+1.39	+0.75	+0.39	+1.65	+1.39	−0.6	+0.16	−1.1	0.5	+/−0.9
Ao/MPA ratio	0.5	0.71	0.73	0.67	0.5	0.38	0.46	0.56	0.6	+/−0.2
MPA/Ao ratio	2	1.4	1.36	1.48	2	2.6	2.1	1.78	1.8	+/−0.4
DA V max [cm/s]	140	114	160	140	130	160	140	150	142	+/−14
Hospital stay [days]	18	13	21	25	22	18	30	50	25	+/−11

**Table 2:** Quantity data of postnatal echocardiographic and clinical findings of cases with a diagnosis of PH. Prenatal a-dispr: prenatal disproportion at the level of atria; Prenatal v-dispr: prenatal disproportion at the level of ventricles; Prenatal 3VV-dispr: prenatal disproportion at three-vessel view; Ao-aortic; TR-tricuspid regurgitation; PR-pulmonary regurgitation; IVS-intraventricular septum; echo-echocardiography; CoA-coarctation of aorta; iv-intravenous; CPAP-continuous positive airway pressure.

	Case 1	Case 2	Case 3	Case 4	Case 5	Case 6	Case 7	Case 8	
Prenatal a-dispr.	+	-	+	+	+	-	+	+	6/8
Prenatal v-dispr.	+	-	+	+	+	+	+	+	7/8
Prenatal 3VV-dispr	+	+	+	+	+	+	+	+	8/8
Ao reversal flow	-	-	-	+	-	+	+	+	4/8
TR	+	+	+	+	+	+	+	+	8/8
PR	+	+	-	+	+	-	-	-	4/8
IVS > 4.5 mm	+	-	+	+	-	-	+	+	5/8
Postnatal echo 1st day CoA	+	+	+	+	+	+	+	+	8/8
Prostin iv 0.01 ug/kg/min (min. 3–5 days)	+	+	+	+	+	+	+	+	8/8
Oxygen, CPAP or respiratory resuscitation	+	+	+	+	+	+	+	+	8/8
Nitric Oxide	-	-	-	-	+	-	+	+	3/8
Sildenafil	-	-	-	-	+	-	+	+	3/8
Autopsy	-	+	-	+	-	+	-	-	3/3

**Table 3:** Factors affecting fetal pulmonary development and PH according to the literature [2,3,4,5,6,7,8,9,10,11,12,13,14,15,16,17,18,19,20].

Maternal	Placental	Fetal	Birth
Use of corticosteroids Selective serotonin-reuptake inhibitor in 3rd trimester Maternal hyperoxygenation Diet rich in polyphenols in late third trimester Vitamin D deficiency Smoking in pregnancy Diabetes mellitus, obesity Pre-eclampsia Chorioamnionitis	Maternal vascular underperfusion (MVU)-fibrinoid necrosis/acute atherosis and distal villous hypoplasia/small terminal villi	Ductus arteriosus constriction, foramen ovale restriction Meconium peritonitis, meconium aspiration syndrome Metabolic mitochondrial alterations in pulmonary artery cells Diaphragmatic hernia RDS-respiratory distress syndrome Pneumonia Sepsis Pulmonary hypoplasia TGA-transposition of the great arteries	Perinatal asphyxia Antenatal hypoxia Gestational weeks <37 or >42 Cesarean delivery

**Table 4:** Comparison analysis research of van Nisselrooij [24] and our analysis.

	van Nisselrooij (2018) [24]	Our Data
Study scope	13 years (2002–2015), 77 fetuses	18 years (2004–2022), 138 fetuses
Diagnostic findings: false positives	31 postnatal (CoA), 46 overall false positives	68 CoA, 70 overall false positives
Diagnostic findings: newborns with PH	10/77	8/70
Echocardiographic features prenatal	Disproportions, CoA suspicion	Detailed: dilation, valve regurgitations, septum hypertrophy, cardiomegaly
Echocardiographic features postnatal	Need for respiratory support	Right ventricular pressure increase, valve regurgitation, serial echo exams
Outcomes: time of delivery	~37–38 weeks	~37–38 weeks
Outcomes: survival	2/10 PH cases survived (spontaneous resolution)	5/8 survived with treatment
Outcomes: deaths	8/10	3/10
Autopsy/comorbidities	No autopsy data; various associated heart defects noted	Confirmation by histopathology; rare severe associated anomalies

**Table 5:** Prenatal and postnatal echocardiographic key features of PAH-pulmonary arterial hypertension [26,27,28,29,30,31].

Prenatal Echocardiographic Key Features of PH:	Postnatal Echocardiographic Key Features of PH:
COA-coarctation of the aorta Cardiomegaly IVS-interventricular septum hypertrophy RV-right ventricle, RA-right atrium, MPA-main pulmonary artery enlargement TR-tricuspid, PR-pulmonary regurgitation Increased PAP = 90 − (0.62 × PAT) and PAS = MFS/PAT; PAT-pulmonary artery acceleration time, PAP-pulmonary artery pressure, MFS-maximal frequency shift of pulmonary artery flow, PAS-pulmonary artery stiffness.	Pulmonary artery pressure (PAP) > 20 mmHg Right ventricular systolic pressure (RVSP) > 40 mmHg Shunts direction across FO and ductus arteriosus (right to left, left to right, or bidirectional) Interventricular septum (IVS) morphology (flattening or bowing of IVS towards the left ventricle) Right ventricular dilation or hypertrophy, systolic and diastolic failure: Decreased: EF-ejection fraction, FAC-fractional area change, PAAT/RVET-pulmonary artery acceleration time/RV ejection time, TAPSE-tricuspid annular plane systolic excursion, MPI-myocardial performance index.

## Data Availability

The data are not publicly available due to EU Data Protection Regulation (GDPR).
